# Supplementary material for: The interactome of intact mitochondria by cross-linking mass spectrometry provides evidence for coexisting respiratory supercomplexes
Source: Mol Cell Proteomics. 2017 Dec 8;17(2):216–32. doi: 10.1074/mcp.RA117.000470 (PMC5795388; doi:10.1074/mcp.RA117.000470)
Supplement: Supplemental Data [file supp_RA117.000470_133922_0_supp_23978_fzffwf.zip › spectra_annotation/mito_DR_spectra_annotation/219-1-14-1-11-1.pdf]

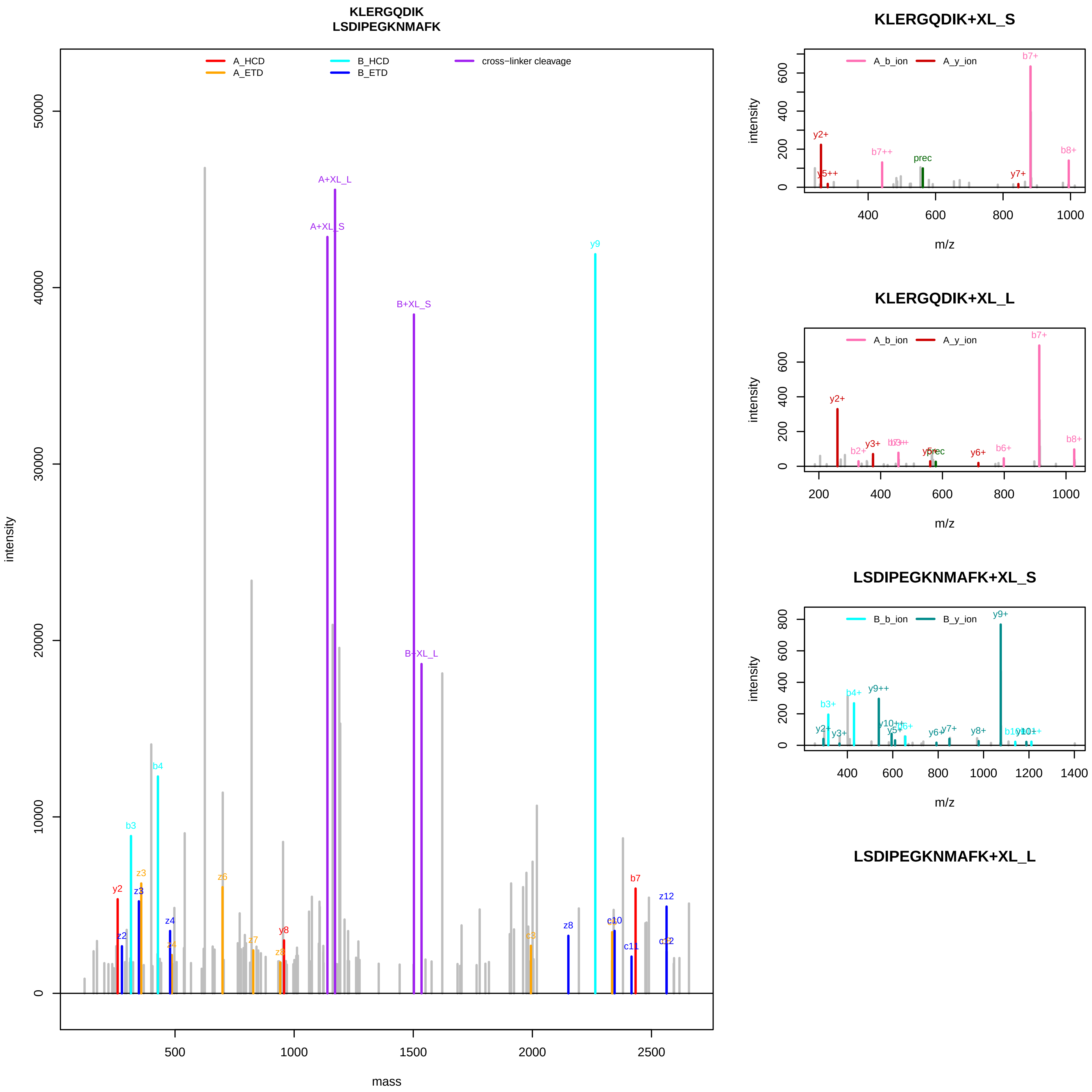
KLERGQDIK
LSDIPEGKNMAFK
A_HCD
A_ETD
B_HCD
B_ETD
cross−linker cleavage
A+XL_L
A+XL_S
B+XL_S
B+XL_L
y9
b4
b3
z3
z6
y2
z4
z2
z7
y8
z8
z8
c3
c10
b7
z12
c11
c12
intensity
mass
0
10000
20000
30000
40000
50000
500
1000
1500
2000
2500
KLERGQDIK+XL_S
A_b_ion
A_y_ion
b7+
y2+
b7++
prec
b8+
y5++
y7+
m/z
KLERGQDIK+XL_L
b2+
y3+
y6+
b6+
LSDIPEGKNMAFK+XL_S
B_b_ion
B_y_ion
y9+
b3+
b4+
y9++
y2+
y3+
y5+
y6+
y7+
y8+
LSDIPEGKNMAFK+XL_L
